# Intra-fraction motion of pelvic oligometastases and feasibility of PTV margin reduction using MRI guided adaptive radiotherapy

**DOI:** 10.3389/fonc.2023.1098593

**Published:** 2023-04-19

**Authors:** Jeffrey Snyder, Blake Smith, Joel St-Aubin, David Dunkerley, Andrew Shepard, Joseph Caster, Daniel Hyer

**Affiliations:** Department of Radiation Oncology, University of Iowa Hospitals and Clinics, Iowa City, IA, United States

**Keywords:** MR-linac, tumor tracking, intra-fraction, adaptive radiotherapy (ART), IMRT (intensity modulated radiation therapy), dose accumulation

## Abstract

**Purpose:**

This study assesses the impact of intra-fraction motion and PTV margin size on target coverage for patients undergoing radiation treatment of pelvic oligometastases. Dosimetric sparing of the bowel as a function of the PTV margin is also evaluated.

**Materials and methods:**

Seven patients with pelvic oligometastases previously treated on our MR-linac (35 Gy in 5 fractions) were included in this study. Retrospective adaptive plans were created for each fraction on the daily MRI datasets using PTV margins of 5 mm, 3 mm, and 2 mm. Dosimetric constraint violations and GTV coverage were measured as a function of PTV margin size. The impact of intra-fraction motion on GTV coverage was assessed by tracking the GTV position on the cine MR images acquired during treatment delivery and creating an intra-fraction dose distribution for each IMRT beam. The intra-fraction dose was accumulated for each fraction to determine the total dose delivered to the target for each PTV size.

**Results:**

All OAR constraints were achieved in 85.7%, 94.3%, and 100.0% of fractions when using 5 mm, 3 mm, and 2 mm PTV margins while scaling to 95% PTV coverage. Compared to plans with a 5 mm PTV margin, there was a 27.4 ± 12.3% (4.0 ± 2.2 Gy) and an 18.5 ± 7.3% (2.7 ± 1.4 Gy) reduction in the bowel D_0.5cc_ dose for 2 mm and 3 mm PTV margins, respectively. The target dose (GTV V_35 Gy_) was on average 100.0 ± 0.1% (99.6 – 100%), 99.6 ± 1.0% (97.2 – 100%), and 99.0 ± 1.4% (95.0 – 100%), among all fractions for the 5 mm, 3 mm, and 2 mm PTV margins on the adaptive plans when accounting for intra-fraction motion, respectively.

**Conclusion:**

A 2 mm PTV margin achieved a minimum of 95% GTV coverage while reducing the dose to the bowel for all patients.

## 1 Introduction

Oligometastatic disease refers to patients with five or fewer lesions and is considered an intermediate disease state between localized and widely metastatic cancer (1, 2). Due to its high precision and steep dose gradients, stereotactic body radiotherapy (SBRT) has proven to be an effective method in treating oligometastatic lymph nodes (3, 4). In these cases, SBRT can improve a patient’s quality of life by delaying or eliminating the need for systemic therapies such as chemotherapy (5). The addition of SBRT in the treatment of oligometastatic disease has also been shown to improve patient overall survival versus standard of care palliative treatment alone (6).

Lymph node SBRT is often delivered in re-irradiation settings where reducing dose to organs at risk (OARs) is critical to minimize potential toxicities. It has been estimated that 40% of patients who receive radiation for a pelvic malignancy will develop a locoregional recurrence in the irradiated field (7). Furthermore, up to 30% of patients who receive SBRT to an oligometastatic lymph node may develop an out of field recurrence in another lymph node potentially requiring re-irradiation (8). These statistics make MRI guided adaptive radiotherapy (MRIgRT) an appealing treatment modality in both the initial and re-irradiation settings for pelvic lymph node oligometastases. MRIgRT provides superior soft tissue contrast and the ability to create daily adaptive plans which account for inter-fraction changes of the target and adjacent OARs (9–11). Additionally, MRIgRT enables noninvasive and nonionizing intra-fraction motion monitoring using either 3D volumetric or 2D cine MR imaging (12, 13). Literature comparing MRIgRT and cone beam computed tomography (CBCT) has shown that fewer OAR constraints were violated by utilizing MRIgRT daily adaptive replanning (14, 15). However, a recent study by Werensteijn-Honingh et al. found that if equivalent PTV margins were used, CBCT provided better bowel sparing as compared to MRIgRT (16). This was primarily due to increased intra-fraction motion in MRIgRT due to longer treatment session times as compared to CBCT treatments on conventional linear accelerators. This highlights the desirability of PTV margin reduction in MRIgRT.

Standard PTV margins for oligometastatic SBRT have been well established for conventional linear accelerators. These margins primarily range from 3 - 5 mm but can extend up to 8mm depending on visibility on CBCT imaging (17–21). With superior soft tissue contrast and the ability to correct for anatomical changes, MRIgRT may enable further margin reduction. However, current literature on MRIgRT SBRT of lymph node oligometastases continue to report using a similar 3 – 5 mm PTV margin (15, 22–26). This is, in part, due to limited published work reporting intra-fraction lymph node motion during extended treatment sessions. Studies that have reported intra-fraction motion of lymph node oligometastases have primarily used pre- and post-3D MRI images, which do not give the position of the target throughout the treatment delivery (21, 27, 28).

Recently, the use of 2D cine MRI imaging coupled with target tracking has been used to determine the required PTV margins and reconstruct the fractional delivered dose to the target for prostate and seminal vesicle treatments (29–31). However, similar studies for pelvic oligometastatic disease are lacking. The aim of this work is twofold: to evaluate the dose delivered to the gross tumor volume (GTV) as a function of PTV margin during MRIgRT of pelvic oligometastases using cine MRI imaging to account for intra-fraction motion; and to compare the dose received by the bowel as a function of PTV margin.

## 2 Materials and methods

### 2.1 Patient selection and clinical workflow

Seven patients with a single pelvic lymph node oligometastasis previously treated using SBRT on an Elekta Unity (Elekta AB, Stockholm Sweden) were enrolled in this study. This research was reviewed by our departmental institutional review board (IRB) and all patients enrolled provided autonomous informed consent which included consent to publish. The study was conducted in accordance with the International Council for Harmonization ICH E6(R2) Good Clinical Practice as adopted by the United States FDA, which aligns with the principles of Helsinki. All patients enrolled in this study had a GTV which was well visualized on a balanced fast field echo (TE 3.8 ms, TR 1.92 ms, flip angle: 40 degrees) cine MRI imaging sequence which comes standard on the Unity system (32).

The patients treated on this study received 35 Gy in 5 fractions to the planning target volume (PTV). Reference planning was conducted on a CT dataset with 2 mm slice thickness in a research version of the Monaco treatment planning system (version 6.01). The lymph node metastasis visualized on the CT image was used for GTV delineation. No additional margin was added to create a CTV (GTV = CTV). For this study, three separate reference plans were made for each patient and fraction consisting of a different PTV margin expansion. The GTV was expanded uniformly by 2mm, 3mm, and 5mm respectively to create the PTV in each plan. A nine-field step-and-shoot IMRT beam arrangement which avoided the cryostat pipe was used for planning (9, 32). Each PTV margin plan was created using a maximum of 10 segment shape optimization loops and IMRT parameters were held constant for each plan. All plans were normalized such that 95% of their respective PTV received 35 Gy. A 1% Monte Carlo per plan statistical uncertainty was used for each optimization.

Online adaptive planning was carried out using the adapt-to shape (ATS) methodology (11, 33). A daily T1 3D image dataset was acquired on the Unity system and contours from the reference CT plan were deformed onto the daily MR image dataset. The OARs and GTV were manually edited as needed. In the clinical setting, the Hyperion optimizer in the Monaco TPS was used to generate a fully adapted plan starting from fluence (34). Following the completion of the adapted plan, online quality assurance procedures were performed and MRI cine motion monitoring images were acquired throughout the duration of treatment (32).

For this study, a simulated ATS workflow was carried out in the research Monaco software. This was done for all 35 fractions (5 fractions per patient) and for each PTV margin such that 105 treatment plans were generated in total. Each plan was normalized for 95% PTV coverage and used the same IMRT parameters as the reference plan. The minimum dose received by the hottest 0.5cc of the bowel (Bowel D_0.5cc_) was compared on each daily adaptive plan as a function of the PTV margin. Violations in all other OAR objectives were assessed using dosimetric criteria previously described for pelvic oligometastases using this dose and fractionation scheme (35).

All the reported OAR doses in this study are from adaptive plans and do not consider effects of intra-fraction motion. The tracking algorithm used in this study only tracked the GTV. Other OAR structures may undergo deformations and/or not move with the same rigid motion as the GTV and thus could not be assessed.

### 2.2 Tracking algorithm

**Figure 1:**
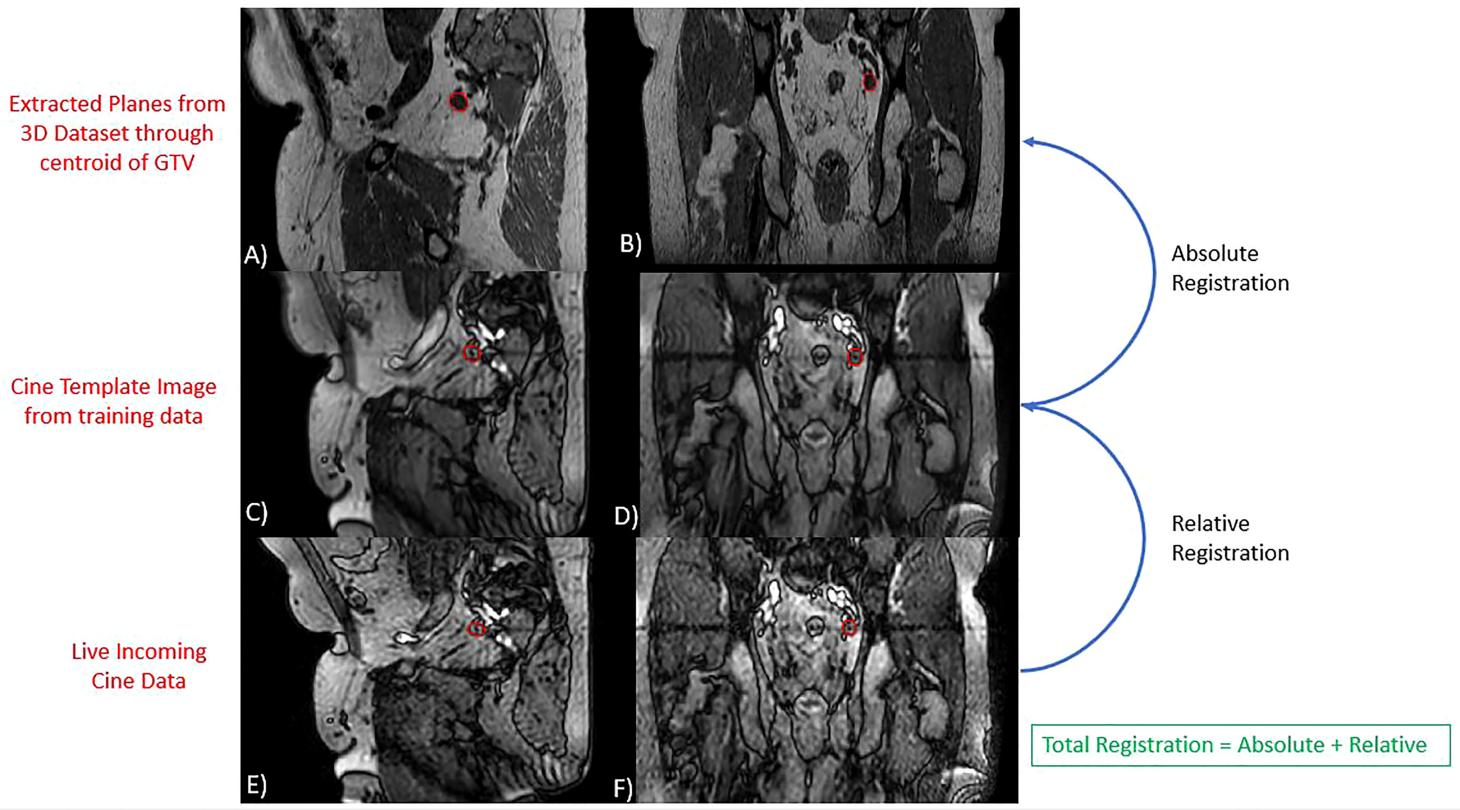
Overview of Tracking Algorithm. Images **(A, B)** represent extracted planes taken through the centroid of the GTV on the daily 3D MRI dataset. Images **(C, D)** are the sagittal and coronal template images generated from the algorithms training phase. Images **(E, F)** are live incoming sagittal and coronal cine motion monitoring images. In each image, the red contour represents the GTV structure contoured during planning **(A, B)** or identified by the tracking algorithm **(C–F)**. The algorithms reported target position is equal to the sum of the relative and absolute registration components.

The GTV was retrospectively tracked on the cine MR images that were acquired during each treatment fraction using a research tracking algorithm provided by Elekta. Details of the algorithm have been previously described (36). Briefly, the tracking process begins with a training phase where 30 sagittal and 30 coronal images are obtained. As this study focuses on tracking targets which do not experience respiratory motion, a single average image from the sagittal and coronal training set is generated. This sagittal and coronal image are referred to as template images. A sagittal and coronal plane through the centroid of the target tracking structure is extracted from the daily 3D MR image dataset that was used for planning. A mutual information algorithm is used to register the template images to the extracted planes of the daily 3D MR image. This component of the registration can be manually edited by the user prior to the initiation of treatment. This is referred to as the absolute registration component. Finally, live incoming cine MRI images are registered to the template image using a cross correlation algorithm which focuses registration on the tracking structure plus an additional margin expansion referred to as the binary mask. The registration of the incoming cine images to the template images is referred to as the relative registration. The total displacement of the GTV is then equal to the sum of the absolute and relative registration components. An overview of the tracking algorithm is provided in **Figure 1**. This algorithm only considers rigid 3D translations and does not account for target rotations or deformations.

To help ensure accurate tracking, the algorithm also employs a quality factor metric. Some of the parameters evaluated by the metric include interplane jitter, detection of large deformations, and large through-plane motion which can alter the apparent size of the target in a given frame. In clinical practice, any frames in which the quality metric reports a failure would result in the radiation beam being turned off. For this reason, any coronal and sagittal paired frames which reported a quality factor failure were excluded from analysis. To assess potential systematic trends in the GTV position with respect to treatment duration, the average position and standard deviation of the GTV over all 35 factions was plotted at each time point recorded.

### 2.3 Intra-fraction dose reconstruction

The delivered fractional dose received by the GTV was reconstructed based on the position of the GTV as determined by the tracking algorithm and the IMRT delivery. To do this, the IMRT delivery time was estimated for each treatment beam such that it could subsequently be synchronized with the MRI cine images. The treatment time per beam was estimated using a kinematic model incorporating beam delivery time and mechanical motion parameters. The Monaco TPS reports the total time required to deliver the monitor units assigned to each beam and reports the total time required to move the MLCs between segments in each beam. To obtain the total delivery time per beam, additional factors were added including the gantry rotation time between each beam angle and a correction factor which accounts for beam on delay and dose rate ramp-up per IMRT segment. Beam on delay refers to the time from which the MLCs reach their intended position until the time that the radiation beam is initiated. A correction factor for beam on delay and dose rate ramp up was determined by comparing Monaco TPS delivery estimates with actual deliveries times for pelvic oligometastatic cases. A correction factor was fitted to minimize the difference between estimated and actual plan delivery times. A value of 1.3 seconds per segment was used in this study.

**Figure 2:**
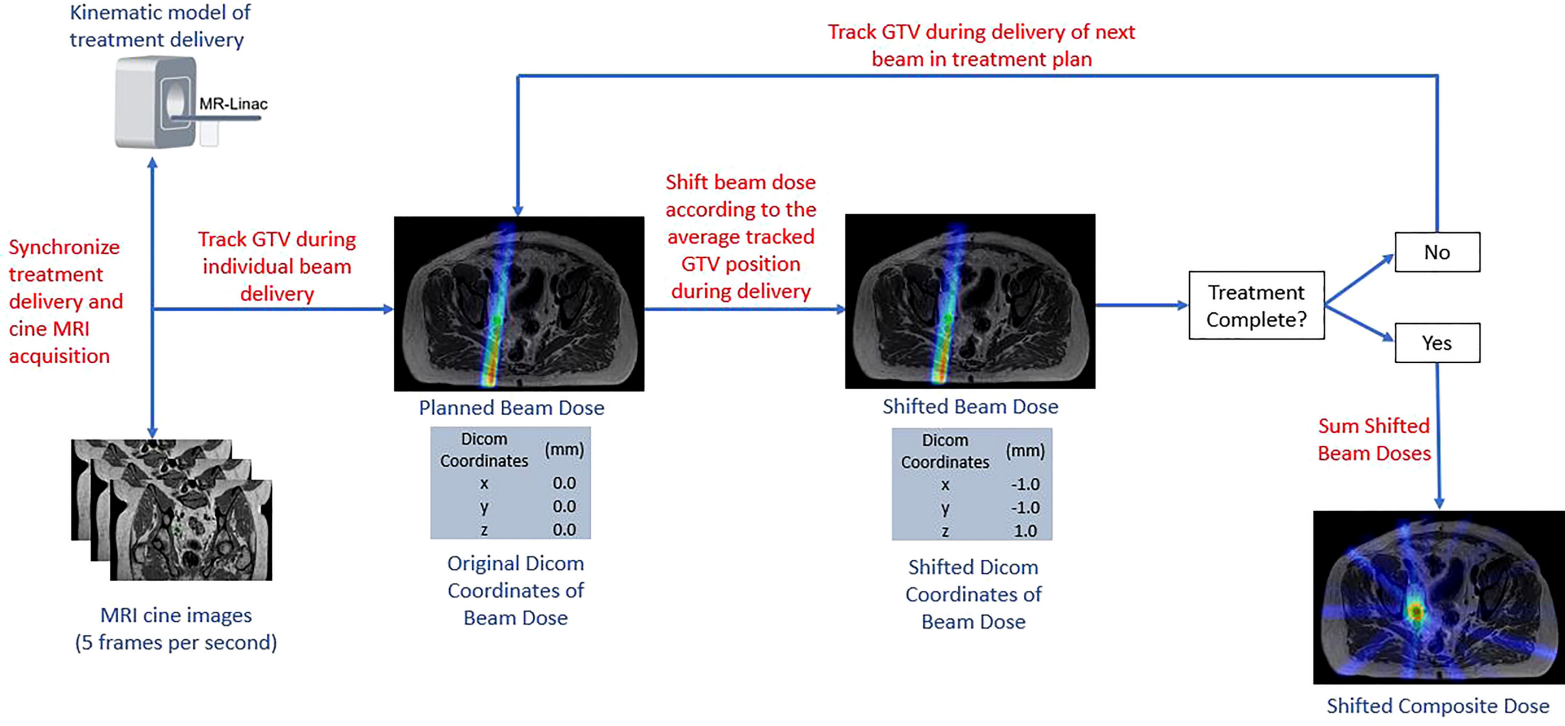
Overview of Intra-fraction Dose Accumulation Workflow.

Dosimetric changes to the GTV coverage were then evaluated for each patient, fraction, and margin using a custom in-house program written in MATLAB (Mathworks Inc, Natick, Massachusetts, Version 2020b). Rigid motion of the GTV was recorded for each cine frame creating a timeline of the GTV motion throughout the course of treatment that was synced with the radiation delivery. The displacement of the GTV from its planned location was taken as the average position during each beam delivery. Radiation dose was exported in a DICOM format. Instead of translating the target within a fixed beam arrangement, the isocenter location listed for each DICOM file was modified to represent the effective motion of the beam from the fixed reference frame of the GTV. Dose distributions were recalculated using a cubic spline interpolation to resample the translated dose distribution from each field back onto the original DICOM coordinate system, which was then compiled into a single radiation dose DICOM file among all modified treatment beam dose distributions. The resulting coverage to the GTV was calculated using Velocity (Varian Medical Systems Inc., Palo Alter, CA, version 3.2.1). A summary of the workflow used to determine the intra-fractional accumulated dose to the GTV is provided in **Figure 2**.

### 2.4 Number of baseline shifts per PTV margin

Future clinical workflows will enable the ability to perform additional intra-fraction plan adaptions *via* a modified adapt-to-position workflow (11, 37). A three second moving average of the tracked GTV position was used to determine how many intra-fraction adaptive interventions would be needed to keep the GTV entirely within the respective PTV margin for each fraction. With this strategy, if any of the three principal directions had a tracked value greater than the PTV margin than an intervention would be required. The time point at which a baseline shift was needed would reset the target position in all three directions back to zero and the relative displacement of the target would be tracked from that point forward to determine if additional baseline shifts corrections would be needed. The number of required baseline shifts was recorded for each PTV margin plan and fraction.

## 3 Results

### 3.1 Dosimetric evaluation

All dosimetric constraints were achieved in 85.7%, 94.3%, and 100.0% of the daily ATS plans which used 5 mm, 3 mm, and 2 mm PTV margins, respectively. All the recorded violations occurred for the Bowel D_.05cc_ < 32 Gy constraint. OAR objectives for the bladder, rectum, and sigmoid were met for all fractions and PTV margins used based on the dosimetric criteria published by Winkel et al. (35) Compared to plans with a 5 mm PTV margin, there was a 27.4 ± 12.3% (4.0 ± 2.2 Gy) and a 18.5 ± 7.3% (2.7 ± 1.4 Gy) reduction in the bowel D_0.5cc_ for 2 mm and 3 mm PTV margins, respectively.

**Figure 3:**
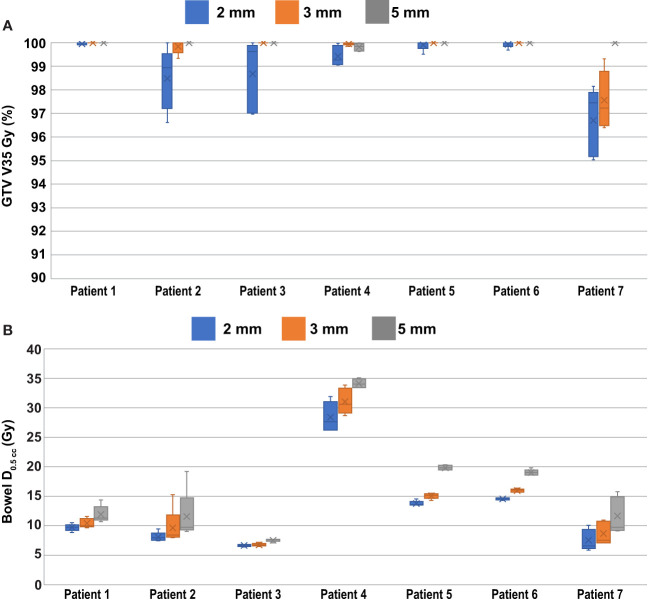
Per Patient Dosimetric Statistics. **(A)** GTV V_35 Gy_ coverage to the PTV when accounting for the effects of intra-fraction motion. **(B)** Bowel D_0.5cc_ doses as calculated on the daily ATS plan.

The percentage of the GTV which was planned to receive at least 35 Gy (GTV V_35 Gy_) on the daily ATS plans was 100.0 ± 0.1%, 100.0 ± 0.1%, and 99.9 ± 0.3%, when averaged among all fractions for the 5 mm, 3 mm, and 2 mm PTV margin plans, respectively. When accounting for the effects of intra-fraction motion, the actual delivered GTV V_35 Gy_ doses were on average 100.0 ± 0.1%, 99.6 ± 1.0%, and 99.0 ± 1.4% for the 5 mm, 3 mm, and 2 mm PTV margin expansion plans. The minimum coverage in any delivered fraction was 99.6% for 5 mm PTV margins, 97.2% for 3 mm PTV margins, and 95.0% using 2 mm PTV margins. The application of baseline shift corrections was not considered in the intra-fraction dose analysis. The box and whisker plot shown in **Figure 3** summarizes the GTV coverage and bowel doses received by each patient and margin combination.

### 3.2 Intra-fraction motion analysis

**Figure 4:**
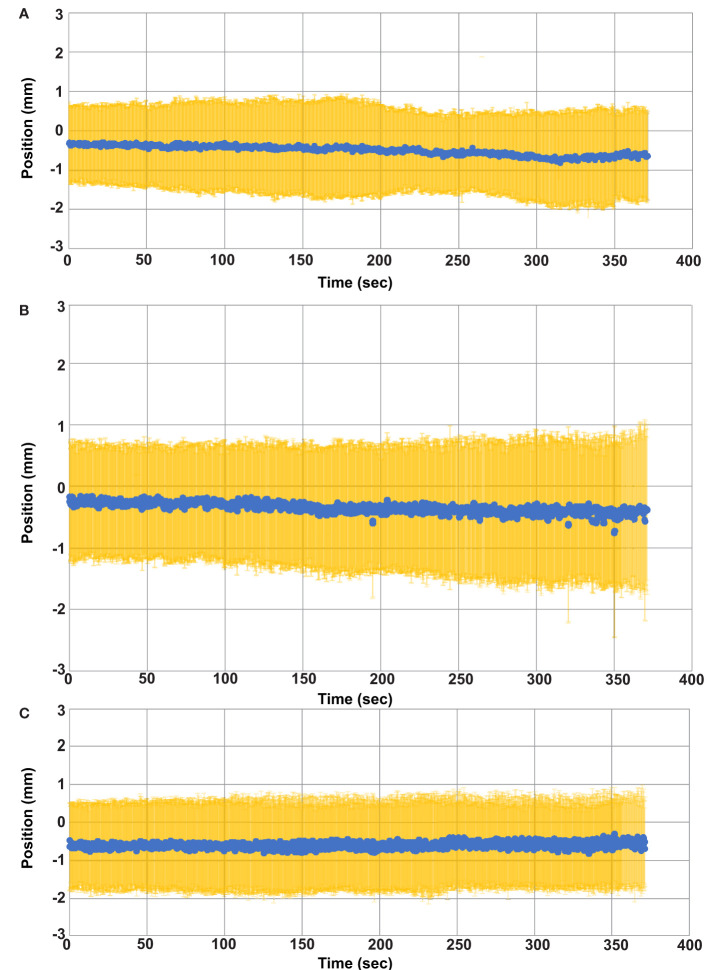
Average GTV Position versus time. The average GTV position (blue) and standard deviation (yellow) for all fractions is plotted as a function of time. **(A)** GTV position in the left (+) and right (-) direction. **(B)** GTV position in the anterior (-) and posterior (+) direction. **(C)** GTV position in the superior (+) and inferior (-) directions.

The quality factor metric indicated successful GTV tracking for greater than 99.8% of the cine imaging frames in each fraction. The average and standard GTV displacement among all patients and fractions is shown in **Figure 4** where each time point represents the average position among all patients and fractions at that same time point during treatment. The average GTV position remained within 1 mm of the planned position in each principal direction for all time points. Thus, the intra-fraction motion for these pelvic oligometastases appeared to be largely random with only a small systematic drift component with respect to treatment duration. While **Figure 4** plots the position of the GTV during treatment, the positional drift of the GTV during adaptive planning was captured using the absolute registration value of the tracking algorithm. This is the reason why the GTV position is not zero at the onset of treatment in **Figure 4**. One of the three principal motion components in the absolute registration exceeded the uniform PTV margin in 17%, 6% and 0% of the delivered fractions for the 2 mm, 3 mm, and 5 mm uniform PTV margins, respectively. An absolute registration value which exceeds the PTV margin indicates that part of the GTV volume extends outside of the PTV prior to the start of treatment. The mean and standard deviation absolute registration values for each patient averaged over all 5 treatment fractions are shown in **Table 1**.

**Table 1:** Patient Intra-Fraction Motion Metrics.

	Average Absolute Registration Value			Number of Baseline Shifts Needed Per Fraction and PTV Margin		
	Left/Right (mm)	Ant/Post (mm)	Sup/Inf (mm)	2mm	3mm	5mm
Patient 1	0.1 ± 0.3	-0.7 ± 0.9	0.5 ± 0.5	0.2 ± 0.4	0.0 ± 0.0	0.0 ± 0.0
Patient 2	0.5 ± 0.8	0.3 ± 0.8	-1.2 ± 0.8	1.0 ± 0.0	0.4 ± 0.5	0.0 ± 0.0
Patient 3	-0.7 ± 1.0	-1.2 ± 0.6	0.6 ± 0.5	0.2 ± 0.4	0.2 ± 0.4	0.0 ± 0.0
Patient 4	-0.2 ± 0.3	-1.4 ± 0.8	0.6 ± 1.0	0.8 ± 0.4	0.2 ± 0.4	0.0 ± 0.0
Patient 5	-0.5 ± 0.5	0.1 ± 1.2	0.2 ± 0.8	0.4 ± 0.5	0.0 ± 0.0	0.0 ± 0.0
Patient 6	0.0 ± 0.7	0.1 ± 0.9	-0.2 ± 0.6	0.4 ± 0.5	0.0 ± 0.0	0.0 ± 0.0
Patient 7	-1.1 ± 1.6	-1.2 ± 1.5	0.3 ± 1.1	2.4 ± 1.7	1.4 ± 0.9	0.0 ± 0.0

**Figure 5:**
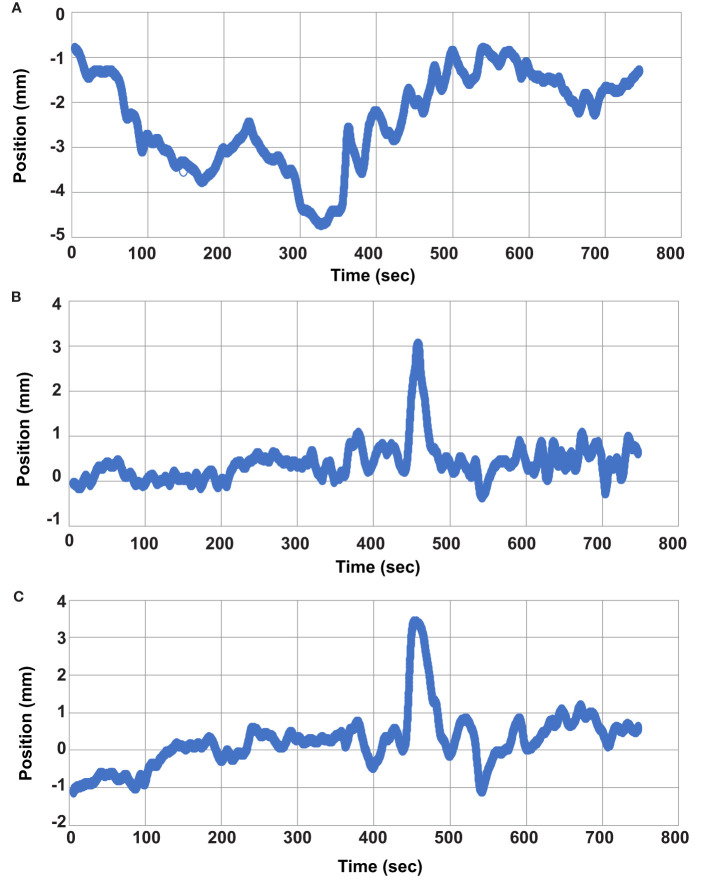
Individual Fraction Motion Trace. Individual motion trace for second treatment fraction of patient 7. **(A)** left/right direction, **(B)** anterior/posterior direction, and **(C)** superior/inferior direction.

Evaluating the GTV position at the beginning and again at the end of treatment would not have adequately captured the maximum range of GTV motion for all cases. **Figure 5** provides one example of this and displays the motion trace of the second fraction for patient 7. The GTV was within 2mm at the start and end of treatment in each of the 3 principal directions, but the target had an excursion of up to 4.7mm during the treatment. The target drifted outside of 2 mm in the lateral direction at approximately 68 seconds and physical patient motion happened causing shifts in the A/P and S/I directions between approximately 440 and 460 seconds. However, the patient returned to a near baseline position by 500 seconds.

### 3.3 Number of baseline shifts per PTV margin

While the average GTV coverage over all 35 fractions was 99.0% or greater, even when using 2 mm PTV margins, there are still instances where a portion of the total volume of the GTV can drift outside of the PTV during treatment causing reduced coverage for individual fractions. **Figure 5** provides one example of this, and the data presented in **Figure 3A** shows that individual fractions can have GTV coverage as low as 95% in this study. The ability to perform intra-fraction baseline shift plans can improve the target coverage in these instances.

Over all fractions the centroid of the GTV never exceeded 5mm in any of the three principal directions and as such no baseline shifts would have been required when using a 5mm PTV margin. An average of 0.8 ± 1.0 and 0.3 ± 0.6 baseline shift corrections per fraction would have been needed when using 2 mm and 3 mm PTV margins, respectively. A maximum of 5 baseline shifts would have been required in a single fraction for the 2 mm margin and up to 3 corrections when using a 3 mm PTV margin. 91.4% and 97.1% of the fractions would have required 1 or fewer baseline shift corrections for 2 mm and 3 mm PTV margin expansions. Only one of the seven patients would have had any fractions requiring more than one baseline shift correction regardless of the margin used. The average number of baseline shifts required for each patient and PTV margin is shown in **Table 1**.

## 4 Discussions

Planning target volumes are added to CTVs to account for random and systematic errors and to ensure appropriate coverage to the CTV due to those uncertainties (38). MRIgRT provides the ability to adapt for inter-fraction anatomical variations and thus may enable PTV margin reductions. However, MRIgRT also requires significantly longer treatment session times as compared to VMAT (34). MRIgRT treatment session times on the order of 60 minutes have been reported and these extended times lead to increased intra-fraction motion (28, 37, 39). One of the main aims of this paper was to assess the practical impact on GTV coverage due to intra-fraction motion in pelvic oligometastasis SBRT treatments using MRIgRT as a function of PTV margin size. Traditional margin recipes such as the Van Herk formula may not be applicable for MRIgRT SBRT treatments due to the small number of fractions and the ability to utilize workflows which mitigate a portion of the intra-fraction motion, such as a baseline shift plan after an initial adaptive plan (28, 30, 40, 41). Recently, Kensen et al. proposed that PTV margins would be considered adequate for rectal MRIgRT patients at the point in which 95% of the primary GTV would receive the prescription dose in 90% of patients (42). Based on this definition, a 2 mm PTV margin expansion would be sufficient for all patients in our cohort as the minimum GTV coverage for all patients and delivered fractions was greater than or equal to 95%. However, if GTV coverages of greater than 97% or 98% are required, then 3 mm and 5 mm PTV margins would be required, respectively.

The clinical ATS workflow used in this study allows physicians to manually edit the GTV if needed, however such manual edits tend to be minor and are not commonly required for pelvic lymph nodes because rigidly aligning the GTV is generally sufficient for this anatomy. There are uncertainties regarding intra-observer and/or inter-observer variability in target delineation in the online setting, however we feel that these are relatively small for pelvic lymph nodes due to them being well visualized with sufficient soft tissue contrast on 3D MRI imaging. Additionally, in conventional radiotherapy target delineation uncertainty on the reference CT scan is a systematic uncertainty while that is not the case in MRIgRT due to the ability to edit the GTV on each fraction, if needed.

Maximum target excursions of up to 4.7mm were measured in this study, which is similar to the maximum value of 5.2 mm reported by Werensteijn-Honingh et al. (28) This study found a median GTV coverage of 100% when using 3mm PTV margins and is equivalent to the value reported by Winkel et al. (21) However, the minimum reported GTV coverage was lower in our study, 96.4% versus 99.7% (21). Winkel et al. evaluated intra-fraction GTV coverage using pre and post MRI imaging. If a similar methodology were used in this study, then the maximum extent of target motion for the fraction exhibiting the lowest coverage would not have been realized. Cine imaging at the beginning and end of treatment showed target positions within 2mm in each of the three principal directions. However, during the delivery of this fraction the target had a maximum excursion of 4.7mm in the left/right direction and greater than 3mm in the anterior/posterior and superior/inferior directions. While using pre and post MRI imaging generally provides a reasonable estimate of the GTV coverage due to intra-fraction motion, this study highlights the limitations of that method. The accumulated intra-fraction dose depends on the extent of excursion of the GTV, and the time points of that excursion with respect to the intensity modulated delivery. This study captures the impacts of those relationships with a higher granularity than previously published studies evaluating MRIgRT for pelvic oligometastases. It also highlights that pre and post MRI imaging may not provide a conservative estimate.

The GTV coverages reported in this work assume that no intra-fraction adaptions were applied. Adaption methods that can apply baseline shift corrections during treatment delivery based on the cine motion monitoring tracking are currently being planned for clinical release. In this study, we found that one or fewer baseline shift corrections would have adequately kept the GTV within a 2mm and 3mm PTV margin in 91.4% and 97.1% of fractions, respectively. We found that a maximum of 5 baseline shift corrections would have been needed for one fraction if 2mm margins were used. While applying this many baseline shifts is possible, it may be impractical from a treatment time perspective. In such cases, a larger PTV margin may be warranted (21, 28). In the future, intra-fraction adaptions may be prepared while still treating which will improve efficiency (37). While this study has shown that 2mm PTV margins acceptably lead to 95% GTV coverage, the fact that the target may extend outside of the PTV in some fractions will likely limit clinical adoption. The impact of such excursions is complex and depends on the volume of the GTV, duration and extent of excursion, and on the IMRT treatment plan. Currently, the true dosimetric impact of these excursions can only be determined retrospectively. However, the application of baseline corrections can prevent such excursions and improve the GTV coverage as compared to treatments delivered without intra-fraction corrections. In the future, methods to reduce treatment session times such as more efficient contouring methods, faster dose optimization, and volumetric modulated arc therapy treatment deliveries are desirable as these will further reduce the number of intra-fraction adaptions needed (34, 43, 44).

While reduction in the PTV margin increases the number of baseline shifts required, it also enables superior OAR sparing as compared to plans with larger margins. This study focused on bowel sparing as that was the main limiting OAR in the online setting. When accounting for the impacts of intra-fraction motion, smaller PTV margins may be required to achieve a practical benefit over CBCT based image guided radiotherapy (16). Smaller PTV margins will improve the feasibility of ultra-hypofractionation techniques where OAR violations are primarily due to overlap with the PTV (35). Reduced margins may also enable improved tumor control probability in settings where SBRT is given as a radiotherapy boost in the pelvis, as compared to conventional fractionation (45). Keeping OAR doses as low as reasonably achievable is important for pelvic oligometastases, which may be treated in re-irradiation settings currently or in the future due to out of field recurrence (7, 8).

One limitation of this study was the small sample size. However, the novel workflow presented gives a more accurate estimate of accumulated intra-fraction dose to the target and can be extended to larger cohorts and/or other anatomical sites. Another limitation is that only patients with well-visualized GTVs in both the sagittal and coronal plane were included. In our experience not all lymph nodes can be adequately visualized on motion monitoring images such that they can be successfully tracked. This highlights a need for the development of cine imaging sequences with different weightings to improve target visualization.

## Data availability statement

The datasets presented in this article are not readily available because the datasets used in this study are not currently available for data sharing. Requests to access the datasets should be directed to jeffrey-snyder@uiowa.edu.

## Ethics statement

The studies involving human participants were reviewed and approved by University of Iowa Institutional Review Board. The patients/participants provided their written informed consent to participate in this study.

## Author contributions

JS and DH -conception, data compilation, writing, and editing. BS – conception, data processing, writing, and editing. JS-A writing and editing. DD writing and editing. AS writing and editing. JC writing and editing. All authors contributed to the article and approved the submitted version. 
